# Long-Term Follow-Up of HRQoL up to Six Years after Outpatient Phase-II Cardiac Rehabilitation

**DOI:** 10.3390/healthcare12030357

**Published:** 2024-01-30

**Authors:** Bianca Auschra, Sebastian Euler, Yara Zehnder, Rubén Fuentes Artiles, David Niederseer, Claudia Zuccarella-Hackl, Roland von Känel, Lena Jellestad

**Affiliations:** 1 Department of Consultation-Liaison Psychiatry and Psychosomatic Medicine, University Hospital Zurich, University of Zurich, 8091 Zurich, Switzerland; 2 Department of Medicine, Limmattal Hospital, 8952 Schlieren, Switzerland; 3 Department of Cardiology, University Hospital Zurich, University of Zurich, 8091 Zurich, Switzerland; 4 Hochgebirgsklinik, Medicine Campus Davos, 7265 Davos Wolfgang, Switzerland; 5 Christine Kühne Center for Allergy Research and Education (CK-CARE), Medicine Campus Davos, 7265 Davos Wolfgang, Switzerland

**Keywords:** HRQOL, cardiac rehabilitation, follow-up, SF-36

## Abstract

**Background:** Low health-related quality of life (HRQoL) is associated with adverse cardiovascular outcomes in coronary heart disease (CHD) patients. Cardiac rehabilitation (CR) improves HRQoL; however, evidence on long-term HRQoL changes after CR, and their predictors, is missing. **Methods:** A total of 153 patients with complete HRQoL data in the short-form (SF)-36 Health Survey at CR entry, discharge and follow-up were included. Using linear mixed-effects regression models for repeated time measurements, we examined predictors of follow-up HRQoL, including age and clinical characteristics. **Results:** Both physical (*t* = −5.66, *p* < 0.001) and mental (*t* = −2.06, *p* = 0.040) HRQoL improved significantly from CR entry to discharge, with improvements remaining stable over a mean follow-up of four years (range 2.4–6.1). Better functional capacity (6MWT) at CR entry predicted better physical HRQoL (*t* = 5.50, *p* < 0.001) and, with a trend, better mental HRQoL (*t* = 1.92, *p* = 0.056) at follow-up. A psychiatric diagnosis at CR entry predicted better mental HRQoL at follow-up (*t* = 3.85, *p* < 0.001). **Conclusions:** Improvements in HRQoL during CR remain stable during long-term follow-up. Levels of functional capacity appear to be relevant to both physical and mental HRQoL at follow-up.

## 1. Introduction

Coronary heart disease (CHD) is the number one cause of global mortality with the second-highest level of disability-adjusted life years (DALY) worldwide [1]. In recent years, the modification of psychosocial risk factors has increasingly come into focus in the prevention and treatment of patients with cardiovascular disease (CHD), ranking third among modifiable cardiovascular risk factors [2]. Health-related quality of life (HRQoL) has hereby proven to be an important and reliable indicator of patients’ perceived health [3]. While objective measures of disease burden such as cardiovascular outcomes are undisputedly important for therapeutic management, maintaining a balanced quality of life (QoL) is equally important for cardiac patients when assessing the impact of disease on everyday live [4]. As poor HRQoL is also an independent risk factor for higher cardiovascular mortality [5] and morbidity [6], its significance as an important outcome measure of therapeutic benefit in cardiac patients has been steadily growing. Cardiac rehabilitation (CR), with its central role in secondary prevention of CHD, improves not only cardiovascular health outcomes [7,8] but also patients’ HRQoL [9]. A large meta-analysis has recently underscored the incremental benefit of CR when compared with usual care for CHD patients in all three dimensions of HRQoL, i.e., emotional, physical and social [10]. Beyond the immediate effects on HRQoL improvement by CR, a recent Cochrane systematic review has highlighted HRQoL improvements in CHD patients in the short-term (6–12 months) follow-up after CR completion [11].

Evidence on the impact of CR on longer-term changes in HRQoL beyond one year following CR is minimal and outdated. Two older studies from 1997 and 2003 demonstrated beneficial effects on HRQoL outcomes in CR patients when compared with non-CR patients up to three years after CR in obese CHD patients [12] and five years after CR completion in patients with coronary artery bypass grafting (CABG) [13]. More recent evidence is lacking, as is evidence on longer-term follow-up of HRQoL in CHD patients beyond 3 years after CR. A better understanding of the long-term health benefits associated with participating in CR is, however, a vital step toward improving the currently low number of CR referrals and utilization rates. Therefore, the aim of this study was to investigate long-term HRQoL changes in CHD patients up to six years after CR completion and to identify potential predictors of HRQoL at long-term follow-up after CR.

## 2. Materials and Methods

**Study design and participants.** In this single-cohort observational study, we assessed n = 311 consecutively admitted CHD patients who completed outpatient phase-II CR at a tertiary care center between January 2016 and September 2019 for long-term changes in HRQoL. In April 2022, we contacted the former CR outpatients by letter, inviting them to participate in a follow-up HRQoL survey. As a measure of HRQoL, we used the Short Form 36 (SF-36) Health Survey, which had already been applied on entry to (T1) and at discharge from (T2) CR. January 2016 marked the beginning of standardized HRQoL measurement using the SF-36, which continued until September 2019, when the assessment tool was replaced. A total of 153 patients were considered for our analysis according to the inclusion criteria, i.e., a diagnosis of CHD and complete data on entry to CR (T1) and follow-up. All subjects gave informed consent to participate in the study. During CR, the cohort received comparable rehabilitative care, which included a twelve-week outpatient CR program with a set of core components defined by the Swiss Working Group for Cardiovascular Prevention, Rehabilitation, and Sports Cardiology (www.scprs.ch, accessed on 8 September 2023) [14]. These components encompassed exercise-based training, smoking and nutritional counseling, as well as psychosocial and psychoeducational interventions. The exercise-based training comprised a standardized 12-week strength and endurance program in 34 sessions of 90 min each. The study was approved by the relevant local authority (2021-02506).

### Variables and Measures

**Health-related quality of life (HRQoL).** We assessed HRQoL using the German adaptation of the Short Form 36 (SF-36) Health Survey. The SF-36 is a validated, self-reported, generic measure of HRQoL which has widely been used with cardiac patients and CR [15,16]. Its 36 items cover eight health subscales of mental (vitality, social functioning, role-emotional, mental health) and physical (physical functioning, role-physical, bodily pain, general health) HRQoL, which are summarized in a Physical Component Scale (PCS, referred to in the following as physical HRQoL) and a Mental Component Scale (MCS, referred to in the following as mental HRQoL) [17]. Scores in each component scale range from 0 to a maximum of 100, with higher scores indicating better HRQoL. We assessed HRQoL at entry to and discharge from CR and at follow-up.

**Exercise capacity.** Exercise capacity during CR was assessed using the 6 min walk test (6MWT) as a valid and broadly applied measure of exercise capacity at submaximal levels [18]. It measures the distance in meters a patient can walk within 6 min. The 6MWT was assessed at entry to and discharge from CR. A change in 6MWT throughout CR was calculated as the ratio of improvement from T1 to T2.

**Psychiatric diagnosis.** The presence of a psychiatric diagnosis was defined as a documented psychiatric disorder (according to the International Statistical Classification of Diseases, tenth version (ICD-10)) in the medical documentation on entry to CR.

**Disease-related variables.** To further characterize the sample of CHD patients, we recorded CHD diagnosis (acute myocardial infarction, unstable angina pectoris and stable angina pectoris), cardiac intervention (percutaneous coronary intervention (PCI), coronary artery bypass surgery (CABG) and valve procedure) and cardiac medication (beta-blocker, ACE-inhibitors, angiotensin receptor–neprilysin inhibitor (ARNI) and SGLT-2 inhibitors). We also documented the ejection fraction at CR entry.

**Statistical analysis.** All information on variables and measures, including sociodemographic and CR-related covariates, was obtained from the electronic medical records. Statistical analyses were performed using R (R Version 4.2.0, RStudio Version 1.2.5019). The primary outcome variables were the physical and mental HRQoL scores at follow-up. Missing data at T2 were imputed via chained equations and predictive mean matching based on the previous timepoint (T1) using the *mice* package [19]. Using the lme4 package [20], we analyzed predictors of physical and mental HRQoL in linear mixed-effects regression models for repeated time measurements (CR entry (T1), CR discharge (T2), follow-up) within individuals. We ran two separate models with physical and mental HRQoL scores at follow-up as the dependent variables and included the following sociodemographic and CR-related factors as potential predictors: age, time since CR discharge, 6MWT at CR entry, change in 6MWT throughout CR (calculated as the ratio of improvement from T1 to T2) and presence of a psychiatric diagnosis at T1. In addition, we controlled for sex, as the proportion of women did not allow for reliable stratified analyses, and the following disease-related factors (as assessed at T1): cardiovascular risk factors (hypertension, dyslipidemia, smoking, obesity (body mass index (BMI) > 30) and diabetes mellitus type II), ejection fraction and Charlson Comorbidity Index (CCI).

## 3. Results

### 3.1. Clinical Characteristics

After exclusion, the final sample comprised n = 153 patients (see Figure S1 in Supplementary Materials depicting a flowchart of participants). Sample characteristics are depicted in Table 1. The majority of the study population was male. Acute myocardial infarction was the main diagnosis leading to CR entry, with most patients having undergone PCI and stenting. Two-thirds of the patients received medication with beta-blockers and ACE inhibitors, while ARNI and SGLT-2 inhibitors were rarely prescribed. Hypertension, dyslipidemia and smoking were the most frequent cardiovascular risk factors. About one in eight patients was diagnosed with a psychiatric disorder at CR entry. Participants showed clinically relevant improvements (>45 m; [21,22]) in their exercise capacity during CR, as measured with the 6MWT. The follow-up time since CR discharge ranged from 2.40 to a maximum of 6.05 years, with an average of 4 years. The group of excluded patients did not differ significantly from the group of included patients in terms of sex, age, presence of a psychiatric diagnosis, the ratio of 6MWT improvement during CR and time since discharge of CR (data not shown). When compared with the group of included patients, the group of excluded patients had lower scores in the 6MWT (*t* = −3.92, *p* < 0.001) at CR entry.

### 3.2. HRQoL Changes over Time

**Figure 1:**
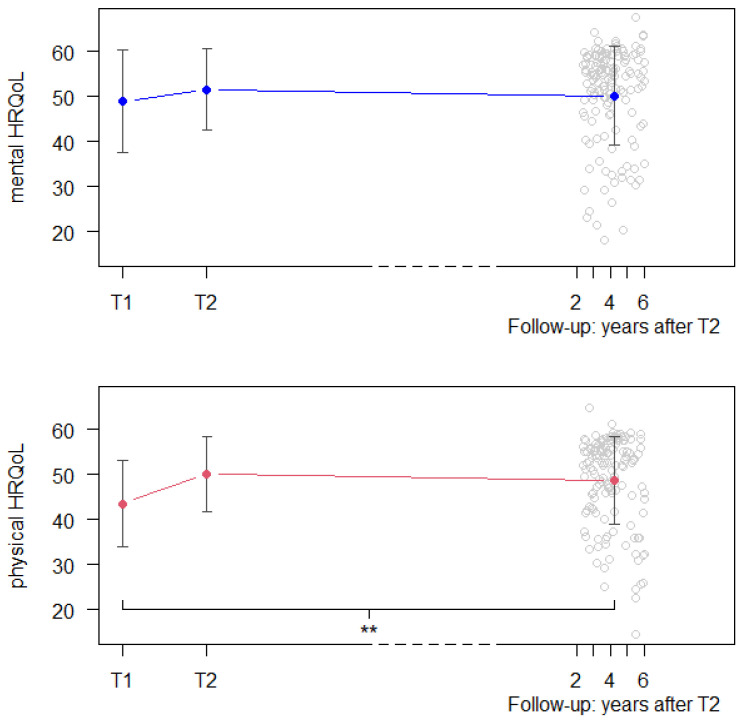
Mean scores and standard deviations of mental and physical HRQoL of the total sample at the three measurement points. ** *p* value < 0.001.

From CR entry (T1) to CR discharge (T2), physical (*t* = −5.66, *p* < 0.001) and mental (*t* = −2.06, *p* = 0.040) HRQoL improved significantly. In both HRQoL subdomains, the improvements remained stable until follow-up. Figure 1 depicts the mean scores of mental and physical HRQoL at the three measurement points (Figure S2 in Supplementary Materials provides additional information on changes in HRQoL in the eight subdomains of the SF-36 survey over time).

### 3.3. Predictors of HRQoL at Follow-Up

In a multiple linear mixed-effects model calculating the associations between sociodemographic, CR- and disease-related variables and physical HRQoL at follow-up, the total model explained 44.0% of the variance (η^2^ = 0.439) in physical HRQoL scores.

A higher score in the 6MWT (*t* = 5.50, *p* < 0.001) at entry to CR emerged as a predictor of better physical HRQoL at follow-up, while a greater improvement in the 6MWT score during CR (higher ratio T2/T1) had a trend of predicting better physical HRQoL at follow-up (*t* = 1.76, *p* = 0.079). In a multiple linear mixed-effects model calculating the associations between sociodemographic, CR- and disease-related variables and mental HRQoL at follow-up, the total model explained 32.9% of the variance (η^2^ = 0.329) in mental HRQoL scores. The presence of a psychiatric disorder at CR entry (*t* = 3.85, *p* < 0.001) emerged as a predictor of better mental HRQoL at follow-up. A higher score in the 6MWT tended to predict better mental HRQoL at follow-up (*t* = 1.92, *p* = 0.056). Table 2 depicts the results of the multiple linear mixed models of fixed effects on HRQoL at follow-up.

## 4. Discussion

In this observational study of 153 CHD patients, we analyzed long-term HRQoL changes and influencing factors after participation in an outpatient CR program. Our analyses demonstrated improvements in HRQoL over the course of CR and stable maintenance of HRQoL levels up to six years after completion of CR. Better physical HRQoL at follow-up was predicted by greater functional capacity at CR entry (measured with the 6MWT) and at trend level by greater improvements in functional capacity during CR. However, despite its increasing importance in measuring the impact of disease on patients’ daily lives, current evidence on long-term HRQoL after CR in CHD patients is lacking. Our study makes a unique contribution to the understanding of how cardiac patients fare in the long term after participation in a CR program.

Similar to previous findings, we observed sustained improvements in markers of quality of life among CHD patients after completing CR. Meta-analytic evidence has recently highlighted the positive effects of participation in a CR program when compared with non-participation on HRQoL during short-term follow-up [23]. However, data on longer follow-up after CR are scarce and highly outdated. To our knowledge, only two studies, both published two decades ago, analyzed post-CR HRQoL at follow-up three (Yu et al. [12]) and five years (Engblom et al. [13]) after CR completion. They compared HRQoL in obese CHD [12] and CABG [13] patients, respectively, with vs. without CR attendance. Participation in CR was associated with better physical [12,13] and mental [13] HRQoL at follow-up. Yu et al. reported significant improvements in HRQoL during CR in all domains of HRQoL measured using the SF-36 survey (though only at the subscale level, with no measures of component scales), which remained largely stable during the 3-year follow-up period in all subscales except social functioning [12]. The program included education classes on prevention and risk factor modification; the physical training comprised a 1–2-week inpatient and an 8-week outpatient exercise program. Beneficial effects were attributed to adopting regular exercise habits that promote functional capacity and psychological wellbeing [12]. The study by Engblom et al. described group differences between CR and non-CR CABG patients over a 5-year follow-up period post-CR. They used the Nottingham Health Profile and self-report questionnaires to assess HRQoL and showed improvement in physical mobility, perceived health and overall life situation in the group of CR patients when compared to non-CR patients at 5-year follow-up [13]. The CR program in this study consisted of four stages, with a central second stage involving 3 weeks of intensive exercise capacity training combined with nutritional counseling and psychoeducational interventions conducted 6–8 weeks after surgery, followed by a concluding 1-day visit 30 months post-surgery. Although the study by Engblom et al. does not provide comparative data on initial HRQoL scores at baseline and after completion of CR and follow-up, it does demonstrate the long-term benefits of CR in cardiac surgery patients. In summary, these previous, albeit old, results covering a shorter follow-up period support our findings of stable HRQoL over time after CR participation and underscore the beneficial effects of CR in this regard even during longer-term follow-up. Our study in a broader CHD patient population, as opposed to limiting it to obese CHD patients [12] or CABG patients [13], provides a broader basis for investigating HRQoL changes in long-term follow-up after CR. The use of the SF-36 mental and physical component scales also allows for better comparability with other (and future) findings on HRQoL long-term follow-up after CR than the use of SF-36 subscales alone or less widely used ratings such as the Nottingham Health Profile.

Earlier evidence revealed that HRQoL of cardiac patients may improve in the longer-term follow-up even without participation in CR. This suggests that part of the improvement in HRQoL may be due to factors outside of CR. However, these findings are inconsistent. Whereas Bahall et al. found improvements in all domains of QoL in patients with first-time myocardial infarction over a 4-year follow-up [24], Hartmann et al. showed worse physical functioning, social functioning and mental health HRQoL during a 10-year follow-up in patients who underwent PCI [25]. In patients after CABG, HRQoL measures improved at 10-year follow-up when compared with HRQoL measures prior to surgery in terms of emotional reaction, pain and energy (assessed with the Nottingham health profile); however, there was no improvement in terms of physical mobility and social isolation [26]. Notably, the scores of all HRQoL subscales decreased between the 5- and 10-year follow-up. In conclusion, there is some evidence that HRQoL in cardiac patients may improve even without CR. However, participation of cardiac patients in CR might enhance this effect. In the direct comparison of HRQoL between patients who underwent CR and those who did not, the benefits seem to be in favor of CR participants. This indicates that CR potentially enhances the long-term trajectories of HRQoL. In our sample, mental HRQoL at CR entry was largely consistent with the previously reported age-adjusted HRQoL reference values of CHD patients [26], whereas physical HRQoL was even higher at CR entry in our sample. Considering the latter in particular, it is even more remarkable that CR not only led to a significant improvement in these values but also maintained their stability throughout the follow-up of period of up to six years after CR. Ultimately, maintaining a balanced HRQoL throughout the course of the disease in spite of the disease-related limitations is central for cardiac patients.

Better mental HRQoL at follow-up was predicted by the diagnosis of a comorbid psychiatric disorder at CR entry and at trend level by higher functional capacity at CR entry. While the relationship between better functional capacity at CR entry and better mental HRQoL scores at follow-up appears rather intuitive, the positive association between the presence of a psychiatric diagnosis and better long-term mental HRQoL seems unexpected. Psychiatric comorbidities in patients with cardiovascular disease are common, and a causal relationship has been suggested [27]. The proportion of comorbid psychiatric disorders in our cohort appears small when compared with the literature showing a prevalence of 15–30% for depression in CHD patients alone [28]. It is reasonable to suspect this to be a problem of underdiagnosis or poor documentation rather than a truly low psychiatric disease burden in our sample, as the prevalence of unrecognized psychiatric disorders in cardiac patients has long been known. The surprising association between a (documented) psychiatric diagnosis at CR entry and better mental HRQoL at follow-up may be due to directing support to this vulnerable patient group, identified as such at CR entry. There is documentation that at least some of the patients were referred for in-hospital psychiatric consultation during or after CR. However, there was no routine specialized psychiatric assessment, which limits the reliability of the psychiatric diagnosis and interpretation of results. Yet in recent years, awareness of the importance of psychosocial risk factors in the treatment of cardiac patients has increased significantly and is now also recognized in European guidelines on cardiac prevention [29]. It can therefore be assumed that psychological counseling was recommended to patients identified as at-risk, which could have contributed to mental stability in the long term. This could provide an explanation for these seemingly surprising results. We encourage further research in this area, including long-term follow-up of these associations after CR.

Sustained physical HRQoL at follow-up was predicted by the level of 6MWT at CR entry. While it may be less surprising that a physical measure of exercise capacity affects physical HRQoL, the 6MWT also emerged as a (marginally significant) predictor of sustained mental HRQoL at follow-up. Surprisingly, in turn, the degree of improvement in the 6MWT from baseline was not associated with sustained HRQoL at follow-up, neither for mental nor physical HRQoL. A previous meta-analysis suggested an association between a gain in 6MWT and improved HRQoL [30], with an improvement > 80m in the 6MWT being predictive of a significant HRQoL improvement during CR. In our study, the mean improvement in the 6MWT was 54m. This could explain why this lower improvement had no predictive potential for HRQoL at follow-up. Moreover, the meta-analysis included studies with a follow-up of at least 6 months, which limits the comparability with our results with a follow-up of up to 6 years.

**Strengths and limitations.** Despite its increasing importance as a treatment outcome in cardiovascular disease, only minimal evidence exists on longer-term follow-up of HRQoL. The major strength of our study was, therefore, the assessment of HRQoL in the long-term follow-up up to six years after CR. The use of the SF-36 survey as a valid and standardized measure of HRQoL supports its comparability with existing evidence. However, some notable limitations must also be taken into account in the interpretation of our results. Although measuring HRQoL in CR patients is nowadays widely recommended, a relevant proportion of patients had to be excluded due to missing HRQoL data during CR. Moreover, due to the long follow-up period after CR, it was not possible to obtain data from all eligible patients due to loss to follow-up. The resultant small sample size hampers the generalizability of our findings. Also, the low proportion of women in the sample did not allow us to conduct reliable analyses stratified by sex. Future studies with larger samples will have to examine whether the longitudinal associations are not moderated by sex. Given the methodological constraints of our study, which by design lacked a control group that did not participate in CR, we also cannot draw conclusions about the underlying causes of HRQoL changes. We cannot exclude that patients may have improved their HRQoL even without their participation in CR, with long-term stable HRQoL. The presence of a psychiatric diagnosis was determined on the basis of a documented psychiatric disorder at CR entry. Possible poor documentation of such a disorder and lack of validation by mental health professionals could limit the validity of the findings in this regard. Given these potential limitations of our results, replication of our findings in larger studies with a prospective design is recommended.

## 5. Conclusions

Our findings provide important evidence of long-term HRQoL changes in CHD patients after CR. The improvements in HRQoL during CR remained stable over the long-term course of up to 6 years after CR. Levels of functional capacity appear to be relevant to both physical and mental long-term HRQoL. In considering that lower HRQoL is associated with worse cardiovascular outcomes, keeping a balanced HRQoL throughout the course of cardiac disease is of major importance. Our results emphasize the central role of CR in cardiac secondary prevention in this regard, both in the short- and long-term course of the disease.

## Figures and Tables

**Table 1:** Clinical characteristics of 153 study participants.

	Total
N	
Total	153
Male	127 (83%)
Female	26 (17%)
Time since CR (years) ^1^	4.00 (1.04)
CHD diagnosis	
Acute myocardial infarction	117 (76.5%)
Unstable angina pectoris	9 (5.9%)
Stable angina pectoris	15 (9.8%)
Presence of arrythmia	19 (12.4%)
Intervention	
PCI/stent	145 (94.8%)
CABG	10 (6.5%)
Valve procedure	4 (2.6%)
Ejection fraction ^1^	54.44 (9.85)
Cardiovascular risk factors	
Hypertension	97 (63.4%)
Dyslipidemia	135 (61.9%)
Smoking	78 (51.0%)
Diabetes mellitus type II	20 (13.1%)
Obesity	31 (20.2%)
Cardiac medication	
Beta-blocker	102 (66.7%)
ACE-inhibitors	98 (64.1%)
ARNI	5 (3.3%)
SGLT-2 inhibitors	7 (4.6%)
Charlson Comorbidity Index ^1^	3.24 (1.76)
Psychiatric disorder	18 (11.8%)
6MWT	
T1 (m) ^1^	539.44 (104.28)
T2 (m) ^1^	593.62 (102.29)
Change ratio T1 to T2 (%) ^1^	11.3 (14.9)

^1^ Mean (SD); CR = cardiac rehabilitation; CHD = coronary heart disease; PCI = percutaneous coronary intervention; CABG = coronary artery bypass graft surgery; ARNI = angiotensin receptor–neprilysin inhibitor; 6MWT = 6 min walking test; m = meters; T1 = baseline score at CR entry; T2 = score at CR discharge.

**Table 2:** Results of a multiple linear mixed model of fixed effects on HRQoL at follow-up (T3).

	Mental HRQOL				Physical HRQoL			
	Estimate *b*	Standard Error	*t*	*p*	Estimate *b*	Standard Error	*t*	*p*
Score T1	−1.32	0.97	−1.37	0.173	−5.01	0.81	−6.23	**<0.001**
Score T2	1.52	0.97	−1.57	0.118	0.98	0.81	1.21	0.226
Psychiatric Disorder	10.87	2.38	4.57	**<0.001**	2.47	2.01	1.23	0.222
Sex	1.11	1.92	0.58	0.564	1.17	1.63	0.72	0.472
Age	−0.02	0.08	−0.22	0.828	0.05	0.07	0.76	0.452
6MWT at T1	0.02	0.01	1.80	**0.075**	0.04	0.01	5.06	**<0.001**
Change ratio T1 to T2	−1.36	5.90	−0.23	0.818	7.78	4.99	1.56	0.122
Time since CR discharge	0.24	0.68	0.35	0.728	−0.45	0.58	−0.78	0.435
Hypertension	1.04	1.41	0.74	0.463	−0.59	1.19	−0.50	0.619
Dyslipidemia	−4.26	1.44	−2.97	**0.004**	0.57	1.21	0.47	0.640
Obesity	−0.99	1.90	−0.52	0.604	−2.10	1.61	−1.31	0.193
Smoking	0.83	1.40	0.60	0.553	−0.38	1.19	−0.32	0.747
Diabetes mellitus type II	3.09	2.74	1.36	0.177	1.06	1.93	0.55	0.583
Charlson Comorbidity Index	1.36	0.56	2.41	**0.017**	−0.21	0.48	−0.45	0.656
Ejection fraction	−0.05	0.08	−0.70	0.485	0.06	0.06	0.95	0.343

HRQoL = health-related quality of life; 6MWT = 6 min walking test; T1 = baseline score at CR entry; T2 = score at discharge of CR; T3 = score at follow-up; *t* = *t*-value. In bold *p* < 0.1

## Data Availability

The data underlying this article cannot be shared publicly due to privacy or ethical restrictions. The data will be shared on reasonable request to the corresponding author.

## Supplementary Materials

The following supporting information can be downloaded at https://www.mdpi.com/article/10.3390/healthcare12030357/s1, Figure S1: Flowchart showing CHD patients. The final sample comprised patients with full data at CR entry (T1) and follow-up (data at CR discharge (T2) imputed from T1 data, where missing). CHD, coronary heart disease; CR, cardiac rehabilitation; Figure S2: Spider diagram depicting changes in mean scores of the eight subdomains of the SF-36 survey across the three measurement time points (CR entry, CR discharge, follow-up). PF, physical functioning; RP, role-physical; BP, bodily pain; GH, general health; VT, vitality; SF, social functioning; RE, role-emotional; MH, mental health.

## References

[1] (2019). Mortality and Global Health Estimates 2019 [Internet]. https://www.who.int/data/gho/data/themes/mortality-and-global-health-estimates.

[2] Yusuf S., Hawken S., Ounpuu S., Dans T., Avezum A., Lanas F., McQueen M., Budaj A., Pais P., Varigos J. (2004). Effect of potentially modifiable risk factors associated with myocardial infarction in 52 countries (the INTERHEART study): Case-control study. Lancet.

[3] Rumsfeld J.S., Alexander K.P., Goff D.C., Graham M.M., Ho P.M., Masoudi F.A., Moser D.K., Roger V.L., Slaughter M.S., Smolderen K.G. (2013). Cardiovascular health: The importance of measuring patient-reported health status: A scientific statement from the American Heart Association. Circulation.

[4] Thompson D.R., Yu C.M. (2003). Quality of life in patients with coronary heart disease-I: Assessment tools. Health Qual. Life Outcomes.

[5] Hofer S., Benzer W., Oldridge N. (2014). Change in health-related quality of life in patients with coronary artery disease predicts 4-year mortality. Int. J. Cardiol..

[6] Conradie A., Atherton J., Chowdhury E., Duong M., Schwarz N., Worthley S., Eccleston D. (2022). Health-Related Quality of Life (HRQoL) and the Effect on Outcome in Patients Presenting with Coronary Artery Disease and Treated with Percutaneous Coronary Intervention (PCI): Differences Noted by Sex and Age. J. Clin. Med..

[7] Price K.J., Gordon B.A., Bird S.R., Benson A.C. (2016). A review of guidelines for cardiac rehabilitation exercise programmes: Is there an international consensus?. Eur. J. Prev. Cardiol..

[8] Anderson L., Oldridge N., Thompson D.R., Zwisler A.D., Rees K., Martin N., Taylor R.S. (2016). Exercise-Based Cardiac Rehabilitation for Coronary Heart Disease: Cochrane Systematic Review and Meta-Analysis. J. Am. Coll. Cardiol..

[9] Jellestada L., Auschraa B., Zuccarella-Hackla C., Principa M., von Känela R., Eulera S., Hermannb M. (2022). Sex and age as predictors of HRQOL change in phase-II cardiac rehabilitation. Eur. J. Prev. Cardiol..

[10] Francis T., Kabboul N., Rac V., Mitsakakis N., Pechlivanoglou P., Bielecki J., Alter D., Krahn M. (2019). The Effect of Cardiac Rehabilitation on Health-Related Quality of Life in Patients with Coronary Artery Disease: A Meta-analysis. Can. J. Cardiol..

[11] Dibben G., Faulkner J., Oldridge N., Rees K., Thompson D.R., Zwisler A.D., Taylor R.S. (2021). Exercise-based cardiac rehabilitation for coronary heart disease. Cochrane Database Syst. Rev..

[12] Yu C.-M., Li L.S.-W., Ho H.H., Lau C.-P. (2003). Long-term changes in exercise capacity, quality of life, body anthropometry, and lipid profiles after a cardiac rehabilitation program in obese patients with coronary heart disease. Am. J. Cardiol..

[13] Engblom E., Korpilahti K., Hämäläinen H., Rönnemaa T., Puukka P. (1997). Quality of life and return to work 5 years after coronary artery bypass surgery. Long-term results of cardiac rehabilitation. J. Cardiopulm. Rehabil..

[14] (2018). Qualitätsanforderungen für die Kardiovaskuläre Prävention und Rehabilitation der SCPRS [Internet]. https://www.scprs.ch/public/richtlinien/richtlinien_deutsch.html.

[15] Brown K. (2003). A review to examine the use of SF-36 in cardiac rehabilitation. Br. J. Nurs..

[16] Smart N.A., King N., Lambert J.D., Pearson M.J., Campbell J.L., Risom S.S., Taylor R.S. (2018). Exercise-based cardiac rehabilitation improves exercise capacity and health-related quality of life in people with atrial fibrillation: A systematic review and meta-analysis of randomised and non-randomised trials. Open Heart.

[17] Ware J., Snoww K., Ma K., Bg G. (1993). SF36 Health Survey: Manual and Interpretation Guide.

[18] Rasekaba T., Lee A.L., Naughton M.T., Williams T.J., Holland A.E. (2009). The six-minute walk test: A useful metric for the cardiopulmonary patient. Intern. Med. J..

[19] van Buuren S., Groothuis-Oudshoorn K. (2011). mice: Multivariate Imputation by Chained Equations in R. J. Stat. Softw..

[20] Bates D., Mächler M., Bolker B., Walker S. (2015). Fitting Linear Mixed-Effects Models Using lme4. J. Stat. Softw..

[21] Shoemaker M.J., Curtis A.B., Vangsnes E., Dickinson M.G. (2012). Triangulating Clinically Meaningful Change in the Six-minute Walk Test in Individuals with Chronic Heart Failure: A Systematic Review. Cardiopulm. Phys. Ther. J..

[22] Holland A.E., Nici L. (2013). The return of the minimum clinically important difference for 6-minute-walk distance in chronic obstructive pulmonary disease. Am. J. Respir. Crit. Care Med..

[23] Taylor R.S., Walker S., Smart N.A., Piepoli M.F., Warren F.C., Ciani O., Whellan D., O’Connor C., Keteyian S.J., Coats A. (2019). Impact of Exercise Rehabilitation on Exercise Capacity and Quality-of-Life in Heart Failure: Individual Participant Meta-Analysis. J. Am. Coll. Cardiol..

[24] Bahall M., Khan K. (2018). Quality of life of patients with first-time AMI: A descriptive study. Health Qual. Life Outcomes.

[25] Hartman E.M.J., Dulfer K., Utens E.M.W.J., van den Berge J.C., Daemen J., van Domburg R.T. (2014). Gender differences in quality of life after PCI attenuate after a 10year follow-up. Int. J. Cardiol..

[26] Herlitz J., Brandrup-Wognsen G., Caidahl K., Haglid M., Karlson B.W., Hartford M., Karlsson T., Sjöland H. (2003). Improvement and factors associated with improvement in quality of life during 10 years after coronary artery bypass grafting. Coron. Artery Dis..

[27] Vaccarino V., Badimon L., Bremner J.D., Cenko E., Cubedo J., Dorobantu M., Duncker D.J., Koller A., Manfrini O., Milicic D. (2019). Depression and coronary heart disease: 2018 position paper of the ESC working group on coronary pathophysiology and microcirculation. Eur. Heart J..

[28] Lichtman J.H., Froelicher E.S., Blumenthal J.A., Carney R.M., Doering L.V., Frasure-Smith N., Freedland K.E., Jaffe A.S., Leifheit-Limson E.C., Sheps D.S. (2014). Depression as a risk factor for poor prognosis among patients with acute coronary syndrome: Systematic review and recommendations: A scientific statement from the American Heart Association. Circulation.

[29] Visseren F.L.J., Mach F., Smulders Y.M., Carballo D., Koskinas K.C., Bäck M., Benetos A., Biffi A., Boavida J.-M., Capodanno D. (2021). 2021 ESC Guidelines on cardiovascular disease prevention in clinical practice: Developed by the Task Force for cardiovascular disease prevention in clinical practice with representatives of the European Society of Cardiology and 12 medical societies with the special contribution of the European Association of Preventive Cardiology (EAPC). Eur. Heart J..

[30] Ciani O., Piepoli M., Smart N., Uddin J., Walker S., Warren F.C., Zwisler A.D., Davos C.H., Taylor R.S. (2018). Validation of Exercise Capacity as a Surrogate Endpoint in Exercise-Based Rehabilitation for Heart Failure: A Meta-Analysis of Randomized Controlled Trials. JACC Heart Fail..

