# Supplementary material for: Long-Term Follow-Up of HRQoL up to Six Years after Outpatient Phase-II Cardiac Rehabilitation
Source: Healthcare (Basel). 2024 Jan 30;12(3):357. doi: 10.3390/healthcare12030357 (PMC10855487; doi:10.3390/healthcare12030357)
Supplement: Supplementary file 1 [file healthcare-12-00357-s001.zip › healthcare-2758677-supplementary.pdf]

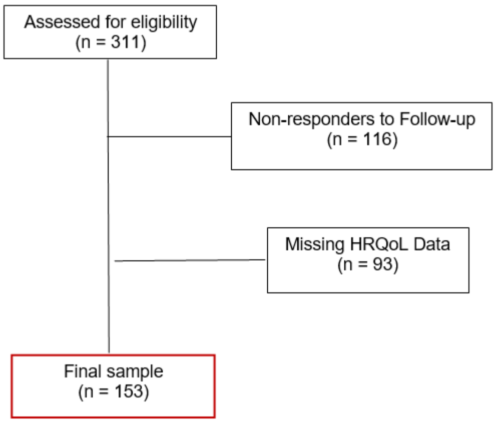

Figure S1. Flowchart showing CHD patients. The final sample comprised patients with full data at CR entry (T1) and follow-up (data at CR discharge (T2) imputed from T1 data, where missing). CHD, coronary heart disease; CR, cardiac rehabilitation.

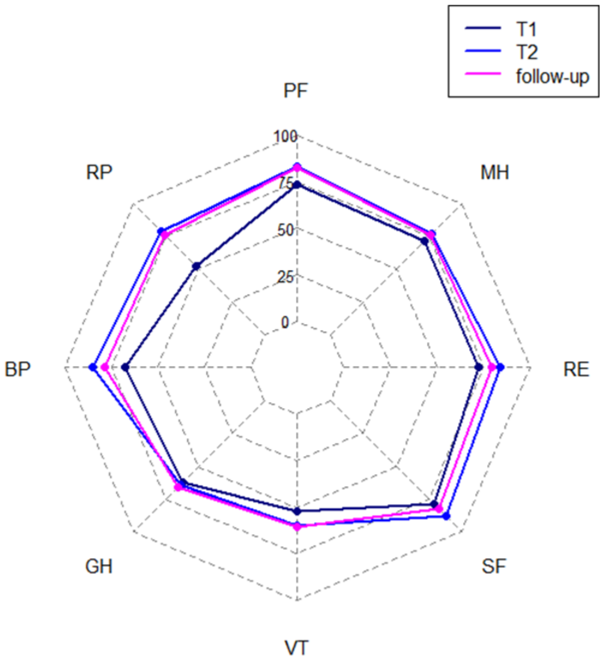

Figure S2. Spider diagram depicting changes in mean scores of the eight subdomains of the SF-36 survey across the three measurement time points (CR entry, CR discharge, follow-up). PF, physical functioning; RP, role-physical; BP, bodily pain; GH, general health; VT, vitality; SF, social functioning; RE, role-emotional; MH, mental health.
